# Association between handgrip strength, balance, and knee flexion/extension strength in older adults

**DOI:** 10.1371/journal.pone.0198185

**Published:** 2018-06-01

**Authors:** Angelica Castilho Alonso, Samia Maria Ribeiro, Natália Mariana Silva Luna, Mark D. Peterson, Danilo Sales Bocalini, Marcos Maurício Serra, Guilherme Carlos Brech, Julia Maria D’Andréa Greve, Luiz Eugênio Garcez-Leme

**Affiliations:** 1 Program in Aging Science, São Judas Tadeu University (USJT), São Paulo, Brazil; 2 Laboratory for the Study of Movement, Institute of Orthopedics and Traumatology, Faculty of Medicine, University of São Paulo (USP), São Paulo, Brazil; 3 State University Vale do Acaraú (UVA), Ceará, Brazil; 4 Department of Physical Medicine and Rehabilitation, University of Michigan-Medicine, Ann Arbor, Michigan, United States of America; University of Padova, ITALY

## Abstract

The objective of the study was to examine the association between handgrip strength (HGS), knee flexion and extension strength, and static and dynamic balance in older women. One hundred and ten women with a mean age of 67.4±5.9 years were assessed for dynamic postural balance using the Time Up & Go Test (TUG) with and without cognitive tasks. Semi-static balance was assessed by means of a force platform; knee flexor and extensor muscle strength was calculated using an isokinetic dynamometer; and HGS using a hand held dynamometer. Weaker HGS was significantly correlated with worse performance in dynamic postural balance, as well as performance with TUG with and without cognitive tasks; however, there was no correlation between HGS and static balance. There was a moderate positive correlation between knee flexion/extension strength and HGS. This suggests that HGS could be used as a proxy indicator of overall strength capacity for clinical screening among older women.

## Introduction

Handgrip strength (HGS) has been widely used as an important indicator of whole body strength. Weak HGS is also considered a predictor of cardiometabolic diseases, disability, morbidity and early mortality [1–3]. A reduction in muscle strength in frail older adults may impair performance in manual tasks, and is associated with other important functional limitations in gait and balance, with significant consequences, such as increased risk for falls and loss of functional independence [4]. Studies indicate an association between decreased HGS with the increasing age, and a correlation between the decrease in muscle mass and muscle strength of the lower limbs [5].

Similar to the decreases in HGS with age, quadriceps weakness is associated with several negative health outcomes in older adults, including functional impairments [6], increased hospital admissions [7] and early mortality [8]. The association between HGS, quadriceps strength, and variables that characterize functional profiles, such as postural balance, may help identify those older adults who require special care [9].

Isokinetic dynamometers and force platforms have been widely used for the assessment of muscle performance and functional capacity, but their high cost reduces feasibility for integrating into the clinical setting. To compensate for difficult access to these types of equipment, simple field tests and assessments have been suggested. For example, HGS is simple and quick to administer, is non-invasive, and does not require large or expensive equipment. Further, when applied correctly, HGS measurement is highly valid and reliable [9,10]. Thus, HGS has been readily used as a general indicator of muscle strength in clinical and population based research [4,9]; however, it is not well established if HGS is highly correlated to other feasible measures of muscle function and balance in older adults.

The objective of this study was therefore to determine the correlation between HGS, knee flexor and extensor muscle strength, and static and dynamic balance in physically inactive older women.

## Materials and methods

This was a cross-sectional study conducted at the Motion Study Laboratory of the Institute of Orthopedics and Traumatology, Hospital das Clinics, Faculty of Medicine, University of São Paulo. All participants gave their written informed consent to participate in this study, which was approved by the Ethics Committee at the Faculty of Medicine, University of São Paulo (registration number 723/2009).

### Study population

One hundred and ten older women over the age of 60 years were assessed for eligibility according to the following inclusion criteria: a) absence of vestibular, proprioceptive, auditory, neurological and/or mental system impairment assessed by questionnaires; b) no complaints of dizziness or lightheadedness; c) no reported use of drugs that may alter postural balance; d) no reported major injury in the lower limbs in the previous six months; e) no reported major surgery that might influence postural balance in the lower limbs and trunk; f) no important limitations of the ankle, knee and hip joint movement; g) presenting normal gait, without limp; h) no existing medical conditions, limitations or deformations in the upper limbs that may alter muscle strength; i) presenting the Mini Mental State Examination (MMSE) or Folstein Test within normal standards for the Brazilian population, that is equal to or above 18/19 points for illiterate older adult people and 24/25 for those with formal education [11]. Volunteers were excluded if, for any reason, they failed to perform any of the postural balance tests or the assessments of muscle strength.

### Assessments

Through an initial interview, the following data were collected: age, height, body mass, body mass index (BMI), and history of illness. Thereafter, the following questionnaires were given: the Mini Mental State Examination (MMSE) [11], and the International Physical Activity Questionnaire (IPAQ version 8 –short form) [12].

### Postural balance assessment

The postural balance assessment (posturography) was performed on a mobile force platform (AccuSway Plus, AMTI, MA, USA). The force platform was connected to a signal-amplifying interface box (PJB-101) that was linked to a computer by means of an RS-232 cable. The data were gathered and stored using Balance Clinic software, configured to a frequency of 100 Hz with a fourth-order Butterworth filter and a cutoff frequency of 10 Hz [13].

All the participants were tested with standardized positioning in relation to the maximum width of the support base (smaller than hip width), with the arms next to the body, and looking straight ahead at a target. The support base was drawn on a sheet of paper in a fixed position on the force platform, corresponding to the anatomical points of distal hallux phalanx, fifth metatarsal head, and lateral and medial malleolus for each foot. Three measurements were made with the eyes open (EO), and three with the eyes closed (EC) for 60 seconds. The arithmetic mean of the results was calculated from the three tests conducted under each condition, and was processed using the Balance Clinic software. The following parameters were used to measure the stability of the participants: medial-lateral and anteroposterior amplitude, the mean velocity calculated from the total displacement of the center of pressure (COP) in all directions (VAvg), and the elliptical area encompassing 95% of the displacement from the COP [14,15].

### Dynamic balance test

TUG with and without cognitive tasks were performed. The goal was to determine how many seconds the individual took to perform the task of getting up from a standard chair (with seat about 46 cm and arms 65 cm high), walking three meters, turning around 180^o^, walking back to the chair, and sitting back down. In the TUG + cognitive tasks (TUG COG) test, the procedure was similar to the traditional TUG, except the participant was required to say the names of animals out loud, as previously described [16].

### Isokinetic dynamometry

Isokinetic dynamometry was used to determine knee extension and flexion strength using the Biodex Multi-Joint System 3 (Biodex MedicalTM, Shirley, NY, USA). The isokinetic dynamometer was calibrated 30-minutes prior to starting the tests. After a standardized warm-up, the participants were positioned for concentric evaluation of extension and flexion movements of the knee joint. Each subject remained seated with the hips at 90° of flexion, and secured to the chair using belts.

The test was started with the dominant limb at a rate of 60°/s. The limb was evaluated by positioning the lateral condyle of the femur in alignment with the mechanical axis of the dynamometer. All the participants performed four submaximal repetitions to become familiar with the equipment, followed by a 60-second rest interval, then two series of five maximal repetitions of knee extension and flexion, starting with the dominant limb. A 60-second standardized rest interval between each series was allowed for all participants. Maximum values obtained from the second series were used for data analysis, in order to prevent a confounding influence of motor learning on clinical isokinetic performance [17]. Constant verbal encouragement was given during the tests in order to promote maximum effort during contractions. The isokinetic variables used were maximum peak torque corrected for body weight (PTQ/BW), and total work (TW).

### Handgrip strength (HGS) test

HGS assessment was performed using a Jamar hand dynamometer, measured in kilograms (kg), using a technique adopted by the American Society of Hand Therapists (ASHT) [10]. The participants were positioned in the manner suggested by Caporrino et al. [18] seated with their back supported, hips and knees flexed at 90°, feet in contact with the ground, elbow flexed at 90°, with forearm and wrist in a neutral position. Participants were then asked to hold the dynamometer and squeeze with as much force as possible. Alternating tests were conducted between right and left hand, followed by a one minute interval between measurements in order to avoid muscle fatigue during the test. Four measurements were performed on each hand. The first was for training and familiarization with the dynamometer and the remaining were used for the HGS assessment, with the results being recorded separately after completion. The mean of the three measurements was used for analyses.

### Statistical analysis

Data were stored and analyzed using SPSS 21.0 software for Windows (SPSS, Inc.) and SAS 9.3 (SAS Institute, Cary, NC). Descriptive analyses including means, standard deviations, medians, minimums and maximums are presented. The Kolmogorov-Smirnov test was used to verify that the continuous variables showed normal distribution, and thereafter the Spearman's correlation test was used to assess the bivariate correlation between measures. Multiple linear regression was used to determine the association between HGS and each of the lower extremity strength and functional outcomes, after adjusting for age and BMI. All regression assumptions were assessed and met for each model. An alpha of 5% was used for all the statistical tests.

## Results

Twenty-three out of the 110 (20.9%) older women were classified according to the IPAQ as irregularly active A, and 87 (79.1%) as irregularly active B. Therefore, none of the participants met the criteria of recommended frequency, intensity, and duration of regular exercise.

Anthropometric characteristics, age and variables related to dynamic and semi-static balance are shown in Table 1.

**Table 1:** Anthropometric characteristics, age and performance of dynamic and semi-static postural balance variables.

	Mean (SD)	Minimum	Maximum
Age (years)	67.4(5.9)	60.0	85.0
Body mass (kg)	71.0(12.8)	36.0	112.0
Height (cm)	156.0(0.6)	136.0	168.0
BMI (kg/m^2^)	29.1(4.8)	18.3	46.6
MMSE	26,8(3.1)	18	30
Number of Drugs	3(2.1)	1	6
**Dynamic balance**			
TUG (s)	9.9(2.5)	5.0	18.5
TUG cognitive (s)	11.8(3.0)	5.8	20.2
**Postural balance (Eyes open)**			
Medial-lateral amplitude (cm)	1.2(0.5)	0.3	3.2
Anteroposterior amplitude (cm)	2.3(0.7)	0.6	4.0
Mean velocity (cm/s)	0.8(0.2)	0.3	2.0
COP Area (cm^2^)	1.8(1.4)	0.1	8.4
**Postural balance (Eyes closed)**			
Medial-lateral amplitude (cm)	1.1(0.7)	-1.4	3.0
Anteroposterior amplitude (cm)	2.1(1.4)	-2.7	5.4
Mean velocity (cm/s)	1.0(0.5)	-2.2	2.0
COP Area (cm^2^)	1.9(1.5)	0.1	8.2

SD–Standard deviation; TUG–Time Up and Go test; COP—Center of pressure; Mini Mental State Examination (MMSE)

There was a negative correlation between HGS and the TUG test, both with and without cognitive task. There was no correlation between semi-static balance and any of the variables (Table 2).

**Table 2:** Bivariate correlation between HGS and dynamic and semi-static balance in older adult women.

	Handgrip (kg)	
	Dominant r(P-value)	Non Dominant r(P-value)
**Dynamic Balance**		
TUG (s)	-0.20 (0.03)*	-0.20 (0.03)*
TUG cognitive (s)	-0.21 (0.02)*	-.028(0.04)*
**Postural balance (Eyes open)**		
Medial-lateral amplitude (cm)	0.08(0.39)	0.07(0.49)
Anteroposterior amplitude (cm)	0.13(0.18)	0.13(0.16)
Mean velocity (cm/s)	0.06(0.51)	0.04(0.69)
COP Area (cm^2^)	0.14(0.16)	0.11(0.26)
**Postural balance (Eyes closed)**		
Medial-lateral amplitude (cm)	0.11(0.27)	0.13(0.01)
Anteroposterior amplitude (cm)	0.07(0.48)	0.09(0.36)
Mean velocity (cm/s)	-0.01(0.92)	-0.04(0.66)
COP Area (cm^2^)	0.11(0.24)	0.12(0.22)

TUG–Time Up and Go test; COP—Center of pressure; r- Spearman (p<0.05).

Knee flexion and extension strength measures were significantly, moderately correlated with HGS (Table 3).

**Table 3:** Correlation between HGS and knee extensors and flexors in older adult women.

**Knee extensor–Dominant**		
PT/BW (%)	0.43 (<0.001)*	0.39 (<0.001)*
Total Work (J)	0.48 (<0.001)*	0.44 (<0.001)*
**Knee flexor–Dominant**		
PT/BW (%)	0.13 (0.21)	0.16 (0.12)
Total Work (J)	0.31 (<0.001)*	0.32 (<0.001)*
**Knee extensor–Non-Dominant**		
PT/BW (%)	0.35 (<0.001)*	0.38 (<0.001)*
Total Work (J)	0.50 (<0.001)*	0.52 (<0.001)*
**Knee flexor–Non-Dominant**		
PT/BW (%)	0.10 (0.36)	0.16 (0.13)
Total Work (J)	0.21 (0.05)*	0.26 (0.01)*

PT/BW—peak torque corrected for body weight; J-joules; r- Spearman (p<0.05).

The association between handgrip strength and TUG and isokinetic strength in older adult women, after adjusting for age and BMI are shown in Table 4.

**Table 4:** Association between HGS and all outcomes in older adult women, after adjusting for age and BMI.

		β	Standard Error	Pr > |t|
**TUG (s)**				
	**HGS**	-0.09	0.05	0.08
	**Age**	0.06	0.05	0.14
	**BMI**	0.14	0.05	0.006
**TUG cognitive (s)**				
	**HGS**	-0.12	0.06	0.07
	**Age**	0;08	0.06	0.17
	**BMI**	0.17	0.06	0.01
**PT/BW (%) Knee extensor–Dominant**				
	**HGS**	1.8	0.56	0.001
	**Age**	-0.68	0.51	0.19
	**BMI**	-2.49	0.56	<0.001
**TOTAL WORK (J)—Knee extensor–Dominant**				
	**HGS**	5.45	1.53	<0.001
	**Age**	-2.80	1.40	0.04
	**BMI**	2.14	1.54	0.17
**PT/BW (%) Knee extensor–Non Dominant**				
	**HGS**	1.64	0.51	0.002
	**Age**	-0.78	0.47	0.10
	**BMI**	-2.73	0.52	<0.001
**TOTAL WORK (J)—Knee extensor–Non Dominant**				
	**HGS**	5.76	1.46	<0.001
	**Age**	-2.48	1.33	0.07
	**BMI**	2.15	1.46	0.14
**PT/BW (%) Knee flexor–Dominant**				
	**HGS**	0.83	0.32	0.01
	**Age**	0.02	0.29	0.94
	**BMI**	-1.47	0.32	<0.001
**TOTAL WORK (J)—Knee flexor–Dominant**				
	**HGS**	2.51	0.85	0.004
	**Age**	-0.52	0.77	0.51
	**BMI**	0.43	0.85	0.61
**PT/BW (%) Knee flexor–Non Dominant**				
	**HGS**	0.44	0.33	0.19
	**Age**	-0.46	0.31	0.13
	**BMI**	-1.68	0.33	<0.001
**TOTAL WORK (J)—Knee flexor–Non Dominant**				
	**HGS**	1.78	0.96	0.07
	**Age**	-1.30	0.88	0.14
	**BMI**	-0.48	0.96	0.62

Legend: PT/BW—peak torque corrected for body weight; J-joules; TUG–Time up Go Test; HGS—Handgrip strength; BMI–Body mass index

## Discussion

The main finding of this study was that HGS was significantly associated with strength of the lower limbs in older adult women. This finding is important because it suggests that HGS could be used as a proxy indicator of overall strength capacity for clinical screening among older women. Moreover, lower HGS was correlated with worse dynamic balance and mobility performance.

Specifically, HGS was positively and moderately correlated to quadriceps and hamstring strength, and thus this measurement represents a measure of global strength, in agreement with den Ouden et al. [19]. Other studies have shown that lower HGS is directly related to impairments of activities of daily living [19], as well as reduced gait speed [1,20]. Such impairments are caused by functional limitations [21], which may arise due to muscle strength deficits in the lower limbs.

Our findings showing a moderate correlation between HGS and isokinetic variables of quadriceps strength (PT/BW, Total Work) is different from the study by Chan et al. [22], wherein they found a weak correlation. In that study, however, they assessed an older population (mean 83 years) with different comorbidities such as cancer, osteoporosis, arthritis and others.

With advancing age, older adults often use more muscle groups of the upper limbs in daily activities. The same does not happen with the muscle groups of the lower extremities, and this is due to the lack of physical activity, which is very common in the older adult population. Chan et al. [22] and Bohannon et al. [23] rationalized this difference as a decreased use of these muscles over time, since older adults are highly sedentary throughout the day, with declining mobility. On the other hand, the study by Fragala et al. [20] demonstrated that both HGS and leg extension strength seem to be suitable for the screening of muscle weakness in older adults. Bohannon [23] found results similar to ours. In that study, HGS and knee extension strength were found to reflect a common functional construct. Therefore, the strength of knee flexors may be adequate to characterize the strength of the lower limbs in older adult people; however, the measurement of HGS may be preferred because it is easier and more practical to incorporate into a clinical setting.

Dynamic balance and mobility, as assessed by TUG and TUG cognitive, were inversely correlated with HGS. Thus, lower muscle strength was correlated with longer duration TUG tests, a finding which corroborates those from other studies [1,23]. Age-related physiological declines occur with diminished neuromuscular and musculoskeletal function, reduced muscle strength, and decreased coordination and motor control. Changes in sensory receptors and peripheral nerves, associated with decreased visual acuity and vestibular function affect postural control and strength production in the lower limbs, leading to a reduction in gait performance, mobility, and consequently, diminished postural balance [14]. It is worth pointing out that the TUG dual-task (i.e., TUG COG) is associated with decreases in executive function, which is important for the selection, planning and coordination of processing tasks, and it is essential for balance control [15]. This finding corroborates those by Taekaema et al. [9] who demonstrated an association between decreased HGS and declines in cognitive capacity. Older adults with reduced capacity tend to be more fragile and have a greater risk of falls [9].

Weak HGS is shown to be predictive of a wide range of negative health outcomes. It is a practical, functional, validated test that is accepted worldwide, even across populations [24]. In the study by Koopman et al. [24], they applied the HGS test in a population characterized as being malnourished and subject to manual labor in Africa. They compared their study sample with a reference sample from a Western culture, and showed that decreases in HGS still occur with increased age, and was still predictive of mortality similar to that seen in Western populations. This reinforces the notion that HGS may be used as a universal biomarker of aging [25]. The results of the present investigation, in a Brazilian elder population, corroborated Koopman [24] findings.

These findings are important for clinical practice, because they represent the value and utility of HGS as a measure of global muscle strength. Moreover, HGS is associated with postural balance, and thus could be used as a simple and low-cost screening metric in the clinical context for determining the general condition of the patient.

## Conclusion

Lower HGS was associated with worse performance in dynamic postural balance, and it is significantly correlated with muscle strength in the lower limbs, suggesting that HGS could be used as a proxy indicator of overall strength capacity for screening among older women.

## Supporting information

S1 Datas (XLSX)
